# Hystero-Epilepsy Complicating Pregnancy

**Published:** 1888-03

**Authors:** C. C. Frederick

**Affiliations:** Assistant in Obstetrics, Niagara University


### *HYSTERO-EPILEPSY COMPLICATING PREGNANCY.**

By C. C. FREDERICK, M. D., Assistant in Obstetrics, Niagara University.

Mr. President and Gentlemen—I think I cannot more profitably occupy your attention than by reporting to you the history of a case which has occurred in my practice within the past eighteen months, viz.: A case of hystero-epilepsy complicating pregnancy.

These cases are rare. The literature of the subject is meagre—in fact, I have not yet found a single reported case. In my search for literature, I came across a paper on "The Hystero-Neurosis," by Dr. George F. Engleman, of St. Louis, in the transactions of the American Gynecological Society of 1877, wherein he describes cases reported by Drs. Fordyce Barker, Louis Mayer, of Berlin, Edgar Holden, of Newark, N. J., and others, together with those observed by himself. These include hystero-psychoses, hystero-neuroses of the eye, pharynx, stomach and bowels, but do not contain descriptions of hystero-epilepsy complicating pregnancy. Engleman says very few have been described. I have not had access to the writings of Charcot and Richer; therefore, if this be so rare a case, I hope it may prove of interest.

* Read before the Buffalo Medical Club, November 23, 1887.

In September, 1886, I was called to see Mrs. S.; *aet.* thirty-two, married, the mother of three children. She was, at this first visit, suffering from one of her convulsive seizures. After she had recovered from it, I obtained from her and her husband the following history: They had recently removed to this city from a city in the southern part of this state. Two and one-half years before, she had been delivered of her last child, a boy. Her pregnancy progressed as usual, till, about a month previous to her labor, she began to have spells of dyspnœa with faintness. These recurred every few days with increasing severity, till, in about two weeks from their commencement, she had a slight convulsion, during which her arms and legs were cramped with severe pain, but she did not lose consciousness. These continued but a few moments. They continued to recur till after her labor. From that date to the time I saw her, a period of two and one-half years, she had been in excellent health, having had only four or five slight seizures at menstrual periods.

This visit was at the time of an expected menstruation, but she did not menstruate. The convulsion was mild, as above described, and readily yielded to chloroform by inhalation.

On further acquaintance with my patient, I found her to be a sensible, active woman, of an even, sunny disposition, not nervous, of slight build, but plump and well nourished, the last person one would suspect of having a hystero-neurosis. But Engleman says: "The hystero-neuroses are generally found in women who can by no means be called hysterical in the sense in which the word is ordinarily used."

Three weeks elapsed from my first visit before she again had a convulsion, more severe. They continued to recur with increasing severity and persistency, till at several times she had them every day for two, three and four days in succession, the number of seizures numbering as high as fifteen or twenty in the two or three hours during which they continued. After a series of convulsions as above described, she would be greatly fatigued and sleep for long periods. There generally was a period of rest for a week, during which time she would regain her appetite, and feel well. I noticed that the convulsions took on a more severe and persistent character coincident with quickening.

Now to describe, as nearly as I can, a series of paroxysms: She always knew, several hours before their appearance, that they were near at hand. The prodromes ordinarily began in the afternoon, but the storm seldom broke in its fury till from seven to ten P. M., and then continued for from two to four hours. The superficial veins of the forehead and backs of the hands began to show engorgement, and her head felt full, the eyes dull. Coincident with the fullness, came dyspnœa and a short, sharp, quick heart-stroke, only a few beats faster than normal. The dyspnœa continued to increase till her chest pumped like a bellows, taking frequent deep inspirations with sighing expirations. Then consciousness would be lost and the spasm would come on, affecting sometimes one lateral half of the body, then the other side, or effecting both at once. The extensor muscles were more uniformly contracted, throwing the head back, the arms above the head lashing the pillows, the fingers widely distended and curved toward the dorsum of the hand. The legs were also more frequently extended and the toes drawn toward the dorsum of the foot.

Occasionally, the position of extreme opisthotonos or pleurothotonos was assumed. In opisthotonos she rested upon her forehead and toes, the body arched to the utmost. During the spasm, inspiration would almost cease, the heart-beat being rapid and pulse feeble. In two to three minutes, the spasm would become milder, inspirations more frequent, and the pulse fuller. As the muscular spasm relaxed, that of the left side was the last to yield. A period of a few minutes would intervene till the next spasm. In this interval, breathing was stertorous, unconsciousness complete. There was no conjunctival reflex, and the pupils were dilated. As before stated, she would have fifteen to twenty of these convulsions, extending over a period of from two to four hours.

How did I treat this case, you ask? I began with the bromides and kept them up in the intervals. Fortunately, she nourished well during the week or ten days, when free from the eclampsia.

At the approach of them, I gave large doses of chloral, but they would come on in spite of it. I gave her all I dared. Her urine was at all times normal, except after the spasms, when she passed large quantities, of low specific gravity. I then resorted to morphine hypodermically, before the seizures, in one-quarter grain doses, but they came on, not so severe, however.

I found that one-quarter grain doses of morphia were too small, and gave one-half grain, and by repeating one-half grain every one-half to three-quarters of an hour till the pupils contracted, the convulsions would not continue more than one and one-half to two hours. During the last few attacks, I did not give the morphia till about time for the spasm to begin, and then injected one grain and then one-half grain in one-half to one hour. During the intensity of the spasm, I gave chloroform till the pulse became soft, and then withdrew it. At the approach of each succeeding spasm, the pulse would again become hard, and then I began chloroform with the effect of shortening each spasm. I foresaw that she would not hold out till the end of gestation, but fought through the seventh month in order to give her child a chance of life. It was her desire, if I thought it possible. The last three weeks of the seventh month drew heavily upon her strength, but she bravely held out to the end. She had arrived at a point then when something must be done at once, and the induction of premature labor was the something to be done. I would not go into the details of the case further, were it not for the unusual difficulty that I experienced in exciting the uterus to action. I will, therefore, briefly as possible, write my experience: I dilated the cervix with Ellinger's dilator sufficiently to allow me to introduce the smallest size Barnes bag. In the course of six hours, I had succeeded in opening the cervix to the size of a fifty-cent piece, no uterine contractions were excited, and the cervical tissues were thick, leathery and resisting. I then introduced the largest size Barnes bag, and holding it in situ by a colpeurynter in the vagina, filled the Barnes bag full. This succeeded in further dilating the cervix slightly, but no pains were excited. About this time, a series of paroxysms came on, and I had to desist from my efforts at premature labor. I was obliged to wait one week before I could begin my efforts again. This time my manipulations were no more successful than before, except that I did excite feeble uterine contractions while the dilators were in the cervix. At this point, I called in Dr. Lothrop, and gave ether and attempted manual dilatation. To attempt to dilate an iron ring, would have been about as easy. I had ruptured the membranes during my attempts at dilatation; the head was at the internal os. By dint of perseverance, a narrow-bladed short forceps was passed through the cervix, the head grasped, and after a half hour of work under ether, the cervix was forced to yield. The child lived a few hours.

The woman made a rapid convalescence, and began to menstruate in one month. The delivery occurred in March, 1887.

At the second, third and fourth menstrual periods, I was called to see her, and found her suffering from the same eclamptic attacks. I learned that, a few days before the period, the usual vascular tension was intensified, and she suffered great distress in her head. The flow continued only for a few hours at a time and scanty. She sometimes menstruated vicariously from the nose, throat, and at one time had pulmonary hemorrhage.

At the fourth period, her convulsions were as severe as any she ever had had, and continued from seven to twelve P. M. I gave her, during that time, two grains of morphia by hypodermic injection.

At the third period, she had partial hemiplegia of the right side, with complete anæsthesia of the same, lasting for about twelve hours.

After the recovery from the fourth period, in July last, I prescribed permanganate of potassium, grains ii, twice daily. At several times during the month, she saw a slight bloody show, and at the regular period in August she felt fullness of the head which all passed away when the flow began, which continued more profusely than normal for her during four days. She now begins the potassium permanganate one week before the expected period, and goes through her menstruation as one in health, without any hystero-neurosis manifesting itself.

